# Impacts of Deficit Irrigation on Photosynthetic Performance, Productivity and Nutritional Quality of Aeroponically Grown Tuscan Kale (*Brassica oleracea* L.) in a Tropical Greenhouse

**DOI:** 10.3390/ijms24032014

**Published:** 2023-01-19

**Authors:** Jie He, Crystalbelle Chang, Lin Qin, Cheng Hsiang Lai

**Affiliations:** 1 Natural Sciences and Science Education Academic Group, National Institute of Education, Nanyang Technological University, 1 Nanyang Walk, Singapore 637616, Singapore; 2 Meod Pte Ltd., 13 Neo Tiew Harvest Lane, Singapore 719838, Singapore

**Keywords:** deficit irrigation, photosynthetic performance, phytochemicals, productivity, Tuscan kale

## Abstract

Tuscan kale was grown aeroponically with 5, 30 and 60 min nutrient spraying intervals (defined as 5 minNSIs, 30 minNSIs and 60 minNSIs). Four weeks after transplanting, some 5 minNSI plants were transferred to a 60 minNSI (5 minNSI → 60 minNSI) and 90 minNSI (5 minNSI → 90 minNSI) for one more week. Significantly lower light-saturated rates of photosynthesis and stomatal conductance were observed for plants grown with a 60 minNSI than with a 5 minNSI. However, all plants had similar internal CO_2_ concentrations and transpiration rates. Reduced light use efficiency but increased energy dissipation was observed in plants grown in a 60 minNSI. A higher nitrate concentration was observed in 60 minNSI plants compared to 5 minNSI and 30 minNSI plants, while all plants had similar concentrations of total reduced nitrogen, leaf soluble protein and Rubisco protein. Plants grown with prolonged NSIs (deficit irrigation) had lower biomass accumulation due to the inhibition of leaf initiation and expansion compared to 5 minNSIs. However, there was no substantial yield penalty in 5 minNSI → 60 minNSI plants. Enhancements in nutritional quality through deficit irrigation at pre-harvest were measured by proline and total soluble sugar. In conclusion, it is better to grow Tuscan kale with a 5 minNSI for four weeks followed by one week with a 60 minNSI before harvest to reduce water usage, yield penalty and enhance nutritional quality.

## 1. Introduction

Tuscan kale (*Brassica oleracea* L.) is a nutritious leafy green vegetable, low in calories but rich in vitamins and essential minerals [1]. Tuscan kale also has antioxidants and cancer prevention potential [2]. Being an incredibly versatile green, Tuscan kale can be eaten raw in salads and smoothies, steamed or roasted into crunchy kale chips. Due to land scarcity, to ensure food security, the Singapore Food Agency encourages local farmers to use agri-food technology to locally produce 30% of Singapore’s nutritional needs by 2030 [3]. In Singapore, nutritious kale is steadily gaining popularity among local farmers as the population moves towards healthy living. The nutritional profile of kale, however, is highly dependent on a variety of growth conditions [4]. Though kale has been widely studied for its nutritional highlights, the effects of growth conditions in a tropical greenhouse and the physiological mechanisms responsible for its productivity and nutritional quality are poorly understood.

Drought, fluctuating temperatures, high solar and ultraviolet radiation, and nutrient deficiency affect productivity and photosynthesis [5,6]. Although a reduction in the productivity of crops grown under stressful conditions presents the main threat to food security, environmental stressors could enhance the production of phytochemicals such as antioxidants as a defense mechanism [7,8]. Due to reduced water uptake, there is limited water to solubilize and transport nutrients such as N, P and K which are used for plant growth [9]. This results in varying degrees of reduction in the dry matter of different plant organs. Usually, shoot biomass accumulation is more affected by drought than root biomass because of the reduction in leaf number and area owing to the loss of turgor [10]. The loss in turgor pressure is linked to reduced stomatal conductance and transpiration rates in drought-stressed plants resulting in photosynthesis declining in multiple ways [11]. Firstly, the reduced leaf number and leaf area can limit photosynthetic area under drought stress [12]. Furthermore, drought stress markedly inhibited photosynthetic gas exchange [8,11]. In response to drought stress, carboxylation is limited when CO_2_ assimilation and leaf internal CO_2_ decreases with stomatal closure to minimize excessive water loss via transpiration. Secondly, the reduction of carboxylation results in excess light energy, which can over-reduce the electron transport chain in the thylakoid membrane. Thus, electrons are used for photorespiration instead of carboxylation to prevent photoinhibition and can lead to the formation of reactive oxygen species (ROS) [13,14]. ROS can cause oxidative damage to the photosynthetic apparatus, impairing enzymes sych as Rubisco, which reduces photosynthesis under drought [8,15]. Thirdly, due to the structural changes in the photosynthetic apparatus, drought can also severely decrease the photosynthetic pigment contents, resulting in a decline in photochemical efficiency [8,15]. The light energy absorbed by chlorophyll (Chl) could transform into Chl fluorescence [16]. The functional state of the photosynthetic apparatus, based on the maximum photochemical efficiency of PSII (F_v_/F_m_ ratio), is a useful physiological indicator to study the susceptibility of plants to drought [15]. Significant decreases in the electron transport rate (ETR) and an increase in non-photochemical quenching (NPQ) of Chl fluorescence are also observed in plants under drought stress [8,17].

In response to drought stress, plants display a range of mechanisms such as changes in root morphology to increase water uptake and nitrate (NO_3_^−^) assimilation [18,19]. In some species, drought has a significant effect on nitrate reductase activity (NRA), possibly due to the reduced uptake and transport of NO_3_^−^, which is tightly linked to water movement, or the lowering of the NR activation state under drought stress [19]. On the other hand, plants usually synthesize various low-molecular-weight osmolytes such as amino acids, sugar and secondary metabolites, and accumulate them for keeping the cell turgid for growth and development during drought stress [20,21,22]. Proline, the key osmolyte protectant has been reported to not only be involved in osmotic adjustment but also in stabilizing proteins, including antioxidant enzymes, and minimizing the damaging effects of ROS [23,24]. It was reported that proline and total soluble sugar (TSS) levels increased in potato leaves under deficit irrigation regimes [25]. In a study with Arabidopsis, although it was reported that soluble sugar showed a greater contribution in osmotic adjustment than proline in response to drought, there is significant interaction between the metabolisms of proline and soluble sugars [26]. Ascorbic acid (ASC) also plays an important role in protecting plant cells from oxidative damage caused by water deficit in plant tissue [27]. Although ASC in all plants exists as a natural antioxidant compound in normal conditions, it would increase under stress conditions [27]. Drought stress also enhances the production of the total phenolic compounds (TPC), another source of natural antioxidants in vegetables [28]. It was also reported that drought stress can enhance crop quality prior to harvest in sugar apples by activating antioxidant activity, ASC and sugar accumulation [22]. Thus, a possible water management strategy to improve crop quality is to induce drought stress through regulated deficit irrigation.

Deficit irrigation, defined as the application of water below the evapotranspiration requirements, can help in conserving irrigation water without compromising vegetative growth and yield [29,30,31]. While there is literature on the effects of deficit irrigation on other crops [29,30,31], limited studies were conducted on Tuscan kale. We hypothesized that although deficit irrigation would affect photosynthetic performance and plant growth, the nutritional quality of Tuscan kale would be enhanced. We would also like to find out if the application of deficit irrigation prior to harvest could enhance the nutritional quality of Tuscan kale without a substantial yield penalty. If proven successful, the amount of water saved can bring substantial benefits to urban farms. To test our hypotheses, the effects of deficit irrigation during growth and pre-harvest on Tuscan kale were studied by varying the nutrient spraying interval (NSI) in aeroponic systems. The findings of this project can broaden our understanding of Tuscan kale in response to drought stress in terms of plant growth, photosynthetic performance, and accumulation of antioxidants. It would also benefit growers, especially commercial farms with aeroponic systems, by enhancing productivity through the efficient use of water and nutrients which can ultimately ensure food security in Singapore.

## 2. Results

### 2.1. Photosynthetic Gas Exchange

**Figure 1:**
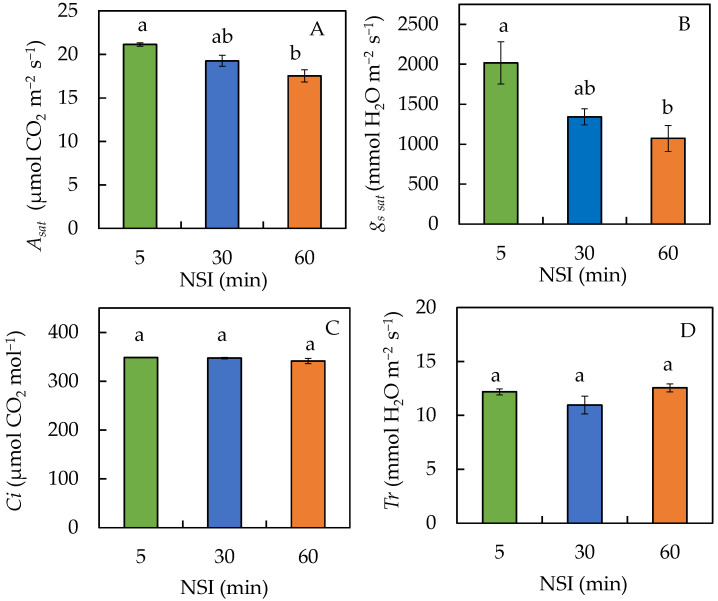
(**A**) Light-saturated rate of photosynthesis (*A_sat_*), (**B**) stomatal conductance (*g_s sat_*), (**C**) internal CO_2_ concentration (*C_i_*) and (**D**) transpiration rate (*T_r_*) of Tuscan kale grown with different NSIs for three weeks after transplanting. Values are means ± standard errors (*n* = 3). Means sharing the same letters are not significantly different from each other (*p* > 0.05) as determined by Tukey’s multiple comparison test.

Significant decreases were observed for the light-saturated rate of photosynthesis, *A_sat_* (Figure 1A), and stomatal conductance, *g_s sat_* (Figure 1B), of plants grown in 60 minNSIs compared to those in 5 minNSIs. *A_sat_* and *g_s sat_* of 60 minNSI plants were 82% and 53% for 5 minNSI plants. However, there were no significant differences in these two parameters between plants grown in 5 minNSIs and 30 minNSIs, and in 30 minNSIs and 60 minNSIs. All plants had similar internal CO_2_ concentration, *C_i_* (Figure 1C) and transpiration rate, *T_r_* (Figure 1D).

### 2.2. Photosynthetic Light-Use Efficiency and Photosynthetic Pigments

**Figure 2:**
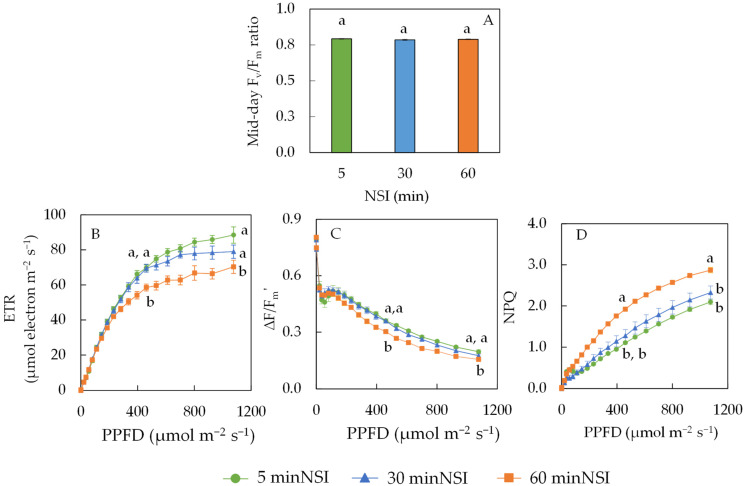
Midday F_v_/F_m_ ratio (**A**), light response curves of electron transport rate, ETR (**B**), effective quantum yield of PSII, ∆F/F_m_’ (**C**) and non-photochemical quenching, NPQ (**D**) of Tuscan kale grown with different NSIs. Values are mean ± standard error (*n* = 5). Means sharing the same letters are not significantly different from each other (*p* > 0.05) as determined by Tukey’s multiple comparison test.

All plants had a similar maximal efficiency of PSII photochemistry (F_v_/F_m_ ratio) measured at midday in the greenhouse regardless of NSIs (Figure 2A). Light response curves of the electron transprot rate, the ETR (Figure 2B), the effective quantum yield of PSII (∆F/Fm’) (Fgiure 2C), and NPQ (Figure 2D) of Tuscan kale grown at different NSIs were measured from the detached leaves in the laboratory. At average and maximum light intensity in the greenhouse of photosynthetic photon flux density, PPFD of 461 µmol m^−2^ s^−1^ and 926 µmol m^−2^ s^−1^, respectively, the ETR and ∆F/F_m_’ of 60 minNSI plants were significantly lower than that of 5 and 30 minNSI plants (Figure 2B,C). For instance, a 60 minNSI resulted in a 17% reduction in the ETR and a 16% reduction in ∆F/F_m_’ measured under a PPFD of 461 µmol m^−2^ s^−1^, compared to those of 5 minNSI plants. A similar trend was also observed for non-photochemical quenching, NPQ (Figure 2D) under both light intensities. However, the NPQ values of 60 minNSI plants were significantly higher than the other two NSI treatments measured under the same light intensity (Figure 2D). The NPQ values of 60 minNSI plants, which were measured at a PPFD of 461 µmol m^−2^ s^−1^, were about 160% in 530 minNSI and 30 minNSI plants.

**Figure 3:**
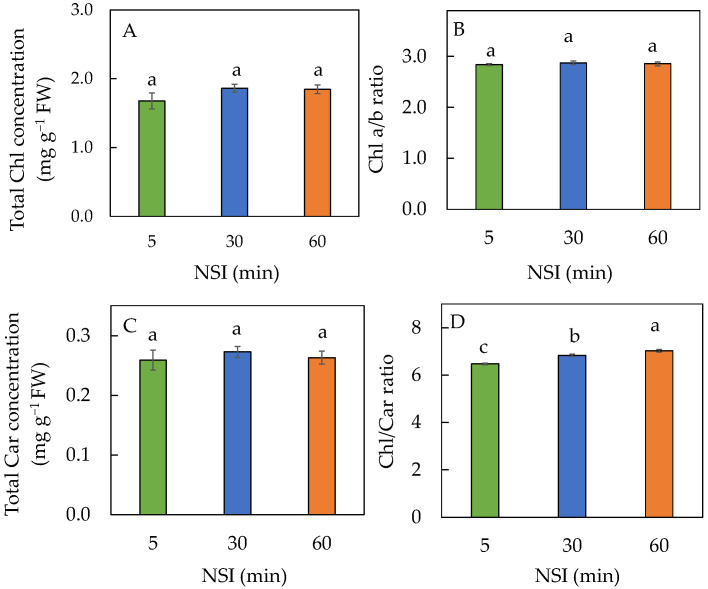
Total Chl concentration (**A**), Chl a/b ratio (**B**), total Car concentration (**C**) and Chl/Car ratio (**D**) of Tuscan kale grown with different NSIs. Values are mean ± standard error (*n* = 5). Means sharing the same letters are not significantly different from each other (*p >* 0.05) as determined by Tukey’s multiple comparison test.

No significant differences were observed for the total chlorophyll (Chl) concentration, Chl a/b ratio and total carotenoids (Car) concentration among the different NSI treatments (Figure 3A–C). For Chl/Car ratio, significant differences were seen among the different NSI treatments, where 60 minNSI plants had the highest Chl/Car ratio followed by 30 and 5 minNSI plants (Figure 3D). The Chl/Car ratios were 7.03, 6.84 and 6.48, for 60, 30 and 5 minNSI plants, respectively.

### 2.3. Nitrate (NO_3_^−^), Total Reduced Nitrogen (TRN), Total Soluble Protein (TSP) and Rubisco Protein

**Figure 4:**
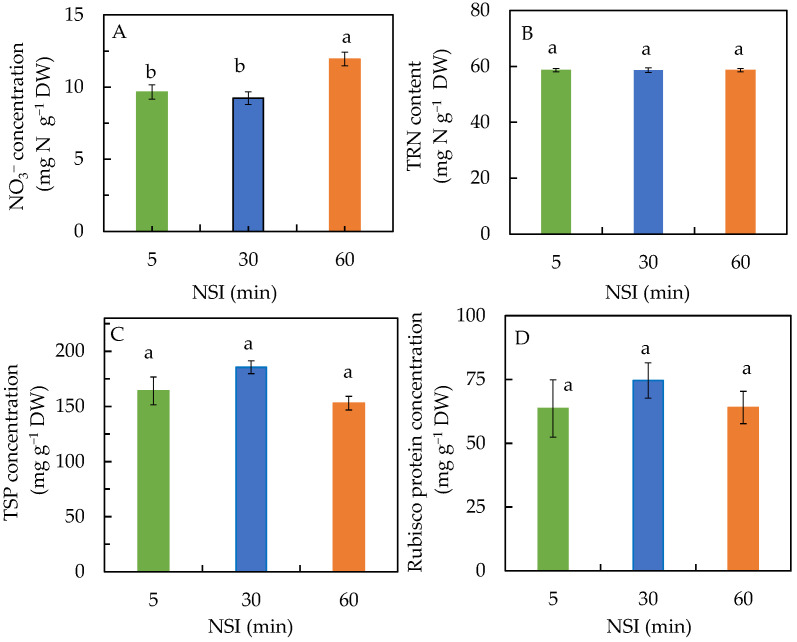
Leaf nitrate, NO_3_^−^ (**A**), total reduced nitrogen, TRN (**B**), leaf total soluble protein, TSP (**C**) and Rubisco protein (**D**) concentrations of Tuscan kale grown at different NSIs for four weeks. Values are means ± standard errors (*n* = 4). Means sharing the same letters are not significantly different from each other (*p >* 0.05) as determined by Tukey’s multiple comparison test.

Leaf NO_3_^−^ concentration of 5 minNSI plants was not significantly different from those of 30 minNSI plants. However, the leaf NO_3_^−^ concentration was significantly higher in 60 minNSI plants than in 5 minNSI and 30 minNSI plants (Figure 4A). It was about 26% higher in 60 minNSI plants compared to those in 5 and 30 minNSI plants. Statistically, all plants had similar levels of TRN, TSP and Rubisco protein (Figure 4B–D).

### 2.4. Leaf Traits, Shoot and Root Productivity and Water Status

**Figure 5:**
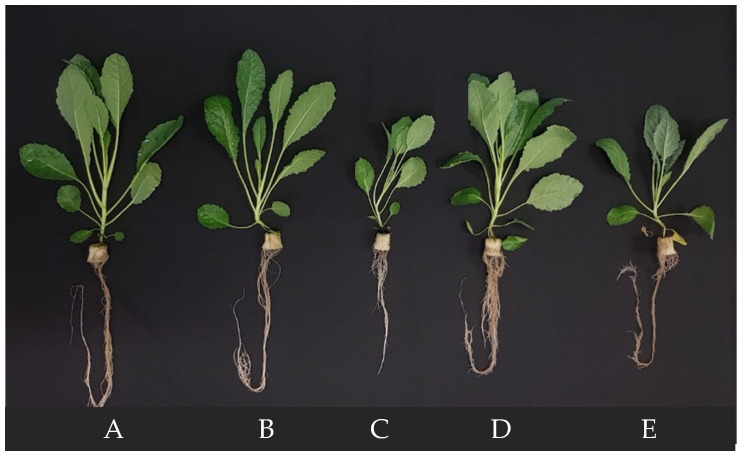
Tuscan kale grown with different NSIs at harvest (5 weeks after transplanting). (**A**) 5 minNSI, 5 weeks; (**B**) 30 minNSI, 5 weeks; (**C**) 60 minNSI, 5 weeks; (**D**) 5 minNSI, 4 weeks → 60 minNSI, 1 week; (**E**) 5 minNSI, 4 weeks → 90 minNSI, 1 week. Refer to Section 4.1 for further details on different NSI treatments.

Figure 5 shows Tuscan kale grown under different NSIs at harvest (5 weeks after transplanting).

**Figure 6:**
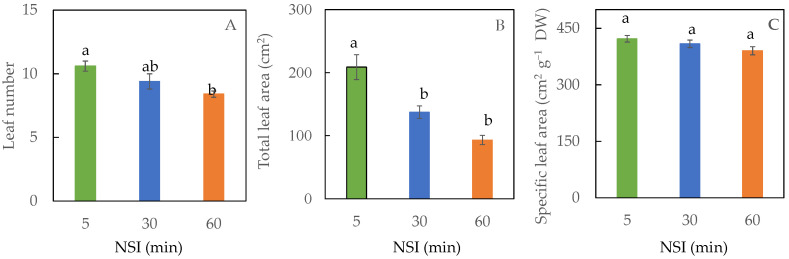
Leaf number (**A**), total leaf area (**B**) and specific leaf area (**C**) of Tuscan kale grown with different NSIs for four weeks. Values are mean ± standard error (*n* = 5). Means sharing the same letters are not significantly different from each other (*p >* 0.05) as determined by Tukey’s multiple comparison test.

The leaf number of 5 minNSI plants was significantly higher than that of 60 minNSI plants (Figure 6A). The 60 minNSI plants had a leaf number of about 79% of 5 minNSI plants. However, there was no significant difference in the leaf number between 30 minNSI plants and the other two treatments. A similar trend was observed for the total leaf area, but the total leaf areas of 30 and 60 minNSI plants were significantly smaller than that of 5 minNSI plants (Figure 6B). Compared to 5 minNSI, prolonging NSI to 60 min resulted in a 53% reduction in total leaf area. No significant differences were observed in the specific leaf area (SLA) among the different NSI treatments (Figure 6C).

**Figure 7:**
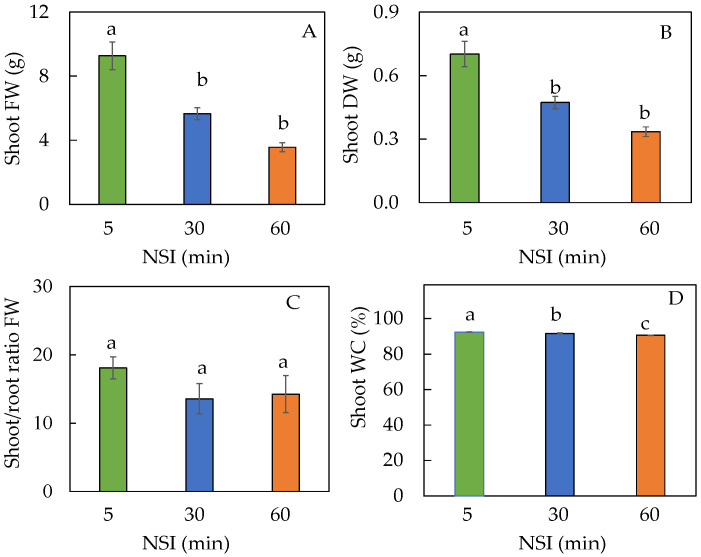
Shoot fresh weight, FW (**A**), shoot dry weight, DW (**B**), shoot/root ratio FW (**C**) and shoot water content, WC (**D**) of Tuscan kale grown with different NSIs for 4 weeks. Values are mean ± standard error (*n* = 5). Means sharing the same letters are not significantly different from each other (*p >* 0.05) as determined by Tukey’s multiple comparison test.

Plants grown in 5 minNSIs were bigger than those grown in 30 minNSIs and 60 minNSIs at harvest (Figure 7A–C), supported by higher shoot fresh weight (FW) and dry weight (DW) (Figure 7A,B). Compared to plants grown in 5 minNSIs, prolonging the NSI from 5 min to 60 min resulted in decreases of shoot FW and DW by 61% and 52%, respectively. However, there were no significant differences in shoot/root ratio FW (Figure 7C) due to the parallel decreases in both shoot FW and DW in plants grown with longer NSIs. Due to the very small standard error, there were statistically significant differences in the shoot water content (WC) among plants grown in different NSIs. However, all plants had shoot WCs greater than 90%, which were 92.3%, 91.6% and 90.6%, in 5 minNSI, 30 minNSI and 60 minNSI plants respectively (Figure 7D).

**Figure 8:**
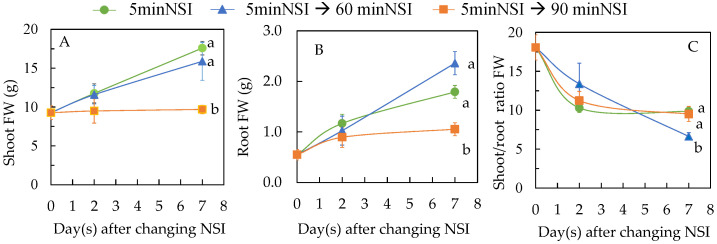
Shoot FW (**A**), root FW (**B**), shoot/root ratio FW (**C**) of Tuscan kale after transferring the 4-week-old 5 minNSI plants to different NSIs. Values are mean ± standard error (*n* = 4). Means sharing same letters are not significantly different from each other (*p >* 0.05) as determined by Tukey’s multiple comparison test.

Figure 8 shows the changes of shoot FW, root FW and shoot/root ratio FW after changing 4-week-old 5 minNSI plants to 60 minNSI and 90 minNSI, respectively, for 2 and 7 days. There was hardly any increase in shoot FW after transferring Tuscan kale grown in 5 minNSIs to 90 minNSIs (5 minNSI → 90 minNSI) during the 7-day transfer period. However, plants transferred from 5 minNSIs to 60 minNSIs (5 minNSI → 60 minNSI) exhibited markedly similar increases in sizes (Figure 5A,D) and shoot FW as those plants remained in 5 minNSIs and had similar shoot FW which were significantly higher than those grown in 90 minNSIs (Figure 8A). For example, 7 days after changing the NSI, the shoot FW of 5 minNSI → 60 minNS plants was 164% of 5 minNSI → 90 minNSI plants. Changes in root FW showed similar trends such as those of shoot FW (Figure 8B). All plants showed decreases in shoot/root ratio FW during the 7-day transfer period. After 7 days of NSI transfer, 5 minNSI → 60 minNSI plants had significantly lower shoot/root ratio FW (Figure 8C), due to the higher accumulation of root biomass (Figure 8B). The shoot/root ratio FW of 5 minNSI → 60 minNSI was 6.64, while the values of 5 minNSI and 5 minNSI → 90 minNS plants were 9.87 and 9.52, respectively.

### 2.5. Nutritional Quality

**Figure 9:**
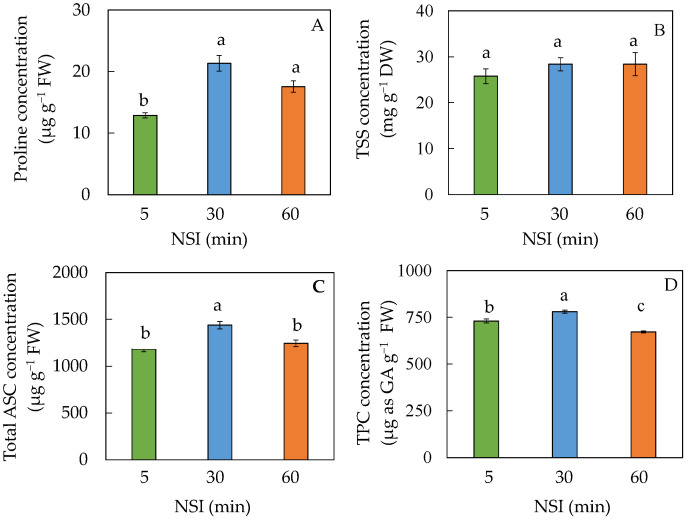
Proline (**A**), total soluble sugar, TSS (**B**), total ascorbic acid, ASC (**C**) and total phenolic compounds, TPC (**D**) concentrations of Tuscan kale grown at different NSIs. Values are means ± standard errors (*n* = 4 for (**A**,**B**,**D**), and *n* = 6 for (**C**)). Means sharing the same letters are not significantly different from each other (*p >* 0.05) as determined by Tukey’s multiple comparison test.

The nutritional quality of Tuscan kale grown under different NSIs was measured by phytochemicals such as proline, TSS, total ASC and TPC. The results are shown in Figure 9.

No significant difference was observed in TSS concentration among Tuscan kale plants grown in different NSIs (Figure 9B). The proline concentration of 5 minNSI plants was significantly lower than those of 30 and 60 minNSI plants (Figure 9A). They were 12.88, 21.34 and 17.56 µg g^−1^ FW for 5, 30 and 60 minNSI plants, respectively. Total ASC concentration in of 30 minNSI plants was significantly higher than those of 5 and 60 minNSI plants (Figure 9C). Similar results were seen in TPC concentration, but 5 minNSI plants had significantly higher concentration than that of 60 minNSI plants (Figure 9D). For example, the total ASC and TPC concentrations in 30 minNSI plants were, 122% and 108% in 5 minNSI plants, respectively.

**Figure 10:**
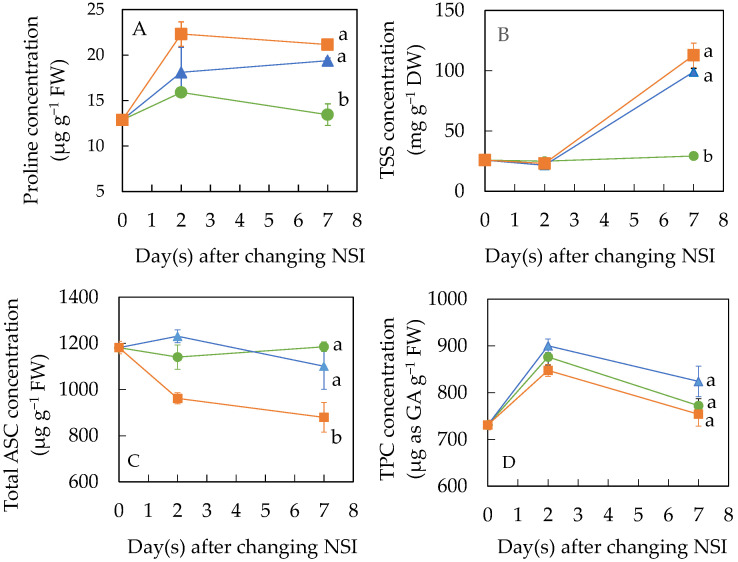
Proline (**A**), total soluble sugar, TSS (**B**), total ascorbic acid, ASC (**C**) and total phenolic compounds, TPC (**D**) concentration of Tuscan kale after transferring the 4-week-old 5 minNSI plants to different NSIs. Values are means ± standard errors (*n* = 4 for (**A**,**B**,**D**), *n* = 6 for (**C**)). Means sharing the same letters are not significantly different from each other (*p >* 0.05) as determined by Tukey’s multiple comparison test.

Accumulations of different phytochemicals were also measured after transferring plants from 5 minNSIs to 60 minNSIs and to 90 minNSIs (Figure 10). Proline concentrations increased 2 days after NSI transfer in all plants with the greatest increase in 5 minNSI → 90 minNSI plants followed by 5 minNSI → 60 minNSI plants. Although there were no further increases in proline concentration from day 2 to day 7, both 5 minNSI → 60 minNSI and 5 minNSI → 90 minNSI plants had similar but significantly higher proline concentrations compared to those plants grown in 5 minNSI after 7 days of NSI transfer (Figure 10A). Proline concentrations were 13.44, 19.40 and 21.15 µg g^−1^ FW, for 5 minNSI, 5 minNSI → 60 minNSI and 5 minNSI → 90 minNSI plants, respectively. There were no significant changes in TSS concentration after 2 days of NSI transfer for all plants. However, after being transferred to longer NSIs for 7 days, TSS concentrations were 3.83- and 3.38-fold higher, respectively, in 5 minNSI → 90 minNSI plants and 5 minNSI → 60 minNSI plants compared to those plants grown in 5 minNSI. High TSS concentration could result in a sweeter-tasting Tuscan kale, which can make it more palatable as kale is known to be bitter. During the 7-day NSI transfer period, 5 minNSI plants had a constantly low level of TSS concentration (Figure 10B). It is interesting to note that the deficit irrigation induced by longer NSIs did not enhance total ASC and TPC concentrations (Figure 10C,D). Instead, total ASC concentration was significantly lower in 5 minNSI → 90 minNSI plants than in 5 minNIS plants, which had similar levels of ASC concentration such as those of 5 minNSI → 60 minNSI plants (Figure 10C). The total ASC concentration of 5 minNSI → 90 minNSI plants was 74% in 5 minNSI plants. During the 7-day NSI transfer period, all plants had similar concentrations of TPC (Figure 10D).

## 3. Discussion

Plant roots are very sensitive to changes in the rhizosphere environment. In our previous study, it was found that drought stress induced by prolonged NSIs inhibited root growth and development of indoor aeroponically grown *Mesembryanthemum crystallinum* (known as ice plant) and reduced its photosynthetic capacity but enhanced its nutritional quality [32]. Thus, it is feasible to grow vegetable crops with prolonged NSI using indoor aeroponic farming systems to enhance nutritional quality at low production costs through water management. Due to water scarcity in Singapore, water optimization for greenhouse cultivation is also an important part of urban agriculture in terms of food security. Several studies have reported that deficit irrigation, which reduces the wastage of irrigation water, could be used to control vegetative growth without yield penalty [29,30,31]. Thus, using aeroponically grown Tuscan kale in a tropical greenhouse, this study tested the effects of deficit irrigation induced by prolonged NSIs on photosynthetic performance, yield and nutritional quality.

### 3.1. Photosynthetic Gas Exchange

The most important physiological response of plants to drought stress is photosynthesis inhibition resulting from both the stomatal and non-stomatal limitations [11,33,34]. In this study, different variables of photosynthetic gas exchange such as *A_sat_*, *g_s sat_*, *C_i_* and *T_r_* were measured at midday on sunny days from the youngest fully expanded leaves which were attached to the stems in the greenhouse. Decreases in *A_sat_* and *g_s sat_* were observed in plants grown in 60 minNSIs compared to those in 5 minNSIs. Statistically, no significant difference was observed in *A_sat_* and *g_s sat_* between 5 minNSI and 30 minNSI plants, and between 30 minNSIs and 60 min NSIs (Figure 1A,B). A significant decrease in *g_s sat_* in 60 minNSI plants could be the first indicator of plant response to drought stress [33,34] supported by the significantly lower shoot WC compared to those grown with 5 minNSI and 30 minNSI plants (Figure 7D). Partial closure of the stomata was reflected in lower *g_s sat_* of 60 minNSI plants (Figure 1B), resulting in a decrease in CO_2_ availability at the chloroplast level, and thus reducing its *A_sat_* [33,34], as stomatal conductance correlates with overall photosynthetic performance [35]. However, in this study all plants had similar *C_i_* (Figure 1C), suggesting that reduced *A_sat_* in 60 minNSI plants was not caused by *C_i_*, but rather by the reduction in photosynthetic light-use efficiency in 60 minNSI plants (to be discussed in the next section). When subjected to drought stress, some species close their stomata, immediately suppressing photosynthetic carbon gain, while others keep their stomata open to maintain carbon gain. Although *g_s_*_*sat*_ of 30 minNSIs and 60 minNSIs were much lower compared to that of 5 minNSIs, they were still considerably high with more than 1000 mmol H_2_O m^−2^ s^−1^. Extremely high *g_s sat_* around 2000 mmol H_2_O m^−2^ s^−1^ in 5 minNSI plants (Figure 1B) could be due to the high frequency of nutrient spray with short NSIs.

Closing of stomata or reducing the stomata aperture could avoid water loss through transpiration [36]. However, in this study, all plants had similar *T*_r_ (Figure 1D). These results were similar to those reported by Zhang et al. [37] in wheat. There was no correlation between stomatal conductance, *g_s_* and transpiration rate, *T*_r_ in wheat during their diurnal variation under high air relative humidity, RH (36.7%), although *g_s_* was significantly correlated with *T*_r_ under lower RH (15.4%) and moderate RH (28.3%). According to Zhang et al. [37], *Tr* was not correlated with *g*_s_ under high air RH but highly correlated with other atmospheric factors such as temperature and photosynthetically active radiation (PAR) in wheat. In this study, measurements of *A_sat_*, *g_s sat_*, *C_i_ and T_r_* were taken with an average RH of 50% ± 3% and an ambient temperature of 38 ± 1 °C, which were similar to the conditions inside the greenhouse. Although the RH drops during the day as temperature rises, the RH inside our tropical greenhouse was as high as 50% when the measurements of *A_sat_*, *g_s sat_*, *C_i_* and *T_r_* were carried out a few hours after dawn. We have previously reported that on a hot sunny day, the ambient greenhouse temperature was around 38 °C while the leaf temperatures of temperate butterhead lettuce (*L. sativa* L. cv Palma) grown under a constant 20 °C root-zone temperature was close to ambient temperature, which was 2 to 4 °C lower than those gown under hot fluctuating ambient temperatures (23 to 38 °C) [38]. In order to moderate leaf temperatures, all plants grown with different NSIs seem to maintain similar *T_r_*. Not only ambient temperature but also leaf temperature could affect the *T_r_* [38,39]. Reductions in both *g_s_* and *T_r_* could increase water use efficiency under drought stress [40]. However, in the present study, transpiration cooling seemed to be a more critical factor for Tuscan kale grown aeroponically with prolonged NSIs under a hot ambient temperature.

### 3.2. Photosynthetic Light Use Efficiency and Photosynthetic Pigments

The evaluation of photosynthetic light-use efficiency has generally been performed by measuring the maximal efficiency of PSII photochemistry (F_v_/F_m_ ratio), ETR, ∆F/F_m_’ and NPQ (Figure 2). On bright sunny days, midday F_v_/F_m_ ratios of close to 0.8 from dark adapted leaves were observed for all plants (Figure 2A). Generally, low availability of CO_2_ in chloroplasts under high light at midday may lead *to* the decrease of photosynthetic electron consumption, causing a dynamic photoinhibition, reflected by reduced F_v_/F_m_ ratios of less than 0.8 [38]. In the present study, none of the leaves measured had dynamic photoinhibition as midday F_v_/F_m_ ratios were all close to 0.8 (Figure 2A). This result further supports the fact that plants grown with prolonged NSIs (30 minNSIs or 60 minNSIs) had considerably high *g_s sat_* of more than 1000 mmol H_2_O m^−2^ s^−1^, which could maintain similar *C_i_* such as that of plants grown with 5 minNSIs (Figure 1B). However, there were significant differences in the ETR, ∆F/F_m_’ and NPQ measured from light-adapted leaves among different plants grown with different NSIs (Figure 2B–D). Measured under average and maximum light intensity in the greenhouse of PPFDs of 461 µmol m^−2^ s^−1^ and 926 µmol m^−2^ s^−1^, respectively, the 60 minNSI plants had significantly lower ETR and ∆F/F_m_’ compared to 5 minNSI and 30 minNSI plants (Figure 2B,C). The ETR and ΔF/F_m_’ of light-adapted leaves are a useful indicator of linear electron transport for overall photosynthesis [16]. Under drought stress, induced by prolonged NSIs under average and maximal PPFD, Tuscan kale reduced its ETR and ∆F/F_m_’ (Figure 2B,C), indicating a reduction in its photosynthetic light-use efficiency during the daytime in the greenhouse. This could explain the decrease in biomass accumulation in drought-stressed 60 minNSI plants as the energy was used for effective dissipation of excess energy instead, which was reflected by higher NPQ (Figure 2D) to protect against leaf water deficit [41]. While there were no significant differences in total Chl and Car concentrations, and Chl a/b ratio among the different NSI treatments, leaf Chl content was slightly higher in 30 minNSI and 60 minNSI plants than in 5 minNSI plants (Figure 3A,B). Higher Chl concentration generally increases photosynthetic light absorption, which is associated with a higher ETR and ΔF/F_m_’ [42]. Maintaining high photosynthetic pigments is an indication of drought tolerance as Tuscan kale was able to maintain the functionality of the photosynthetic machinery under drought stress [43]. Significantly higher Chl/Car ratios due to the higher Chl concentration were more readily observed in 30 minNSI and 60 minNSI plants than in 5 minNSI plants. However, all plants had similar Car concentrations. The investment in Car (Figure 3C) could be a plant cellular defense mechanism as Car are non-enzymatic antioxidants that dissipate excess excitation energy as heat [43], which was supported by the highest NPQ in 60 minNSI plants (Figure 2D).

### 3.3. Nitrogen (N) Metabolism and Rubisco Protein

Plants require large amounts of N, which is an essential component of most biomolecules. The majority of reduced N in plants is invested in photosynthetic machinery and is thus a significant contributor to plant biomass [44]. In this study, no significant effects on NO_3_^−^ uptake were found in plants with longer NSIs, as there were no significant differences in leaf NO_3_^−^ concentration between 5 minNSI plants and 30 minNSI plants (Figure 4A). The 60 minNSI plants even had significantly higher NO_3_^−^ concentrations than 5 minNSI and 30 minNSI plants. In this study, prolonged NSIs seemed similar to long-term drying-rewetting drought treatments [45] that had no effect on plant N concentrations (Figure 4B). It could also be due to their slow growth and small size. Thus, prolonged NSIs did not result in N deficiency in Tuscan kale plants. This was also supported by the fact that the adequate tissue level of N required by plants is around 1.5% according to Epstein [46]. In this study, all Tuscan kale plants had similar TRN concentrations, which were about 5.9% (Figure 4B). The results of this study were also similar to our previous study with ice plants grown under prolonged NSI conditions [32]. Ice plants grown with prolonged NSIs also had adequate shoot TRN, which was greater than 2% [32]. Both Chl and protein such as TSP and Rubisco protein are possible ways for plants to store N [47]. This may explain the similar or higher Chl (Figure 3A), TSP (Figure 4C) and Rubisco (Figure 4D) concentrations in 30 minNSI and 60 minNSI plants compared to those of 5 minNSI, although there were no significant differences in these parameters among the different plants. These results also suggest that reduced *A_sat_* in 60 minNSI plants (Figure 1A) did not correspond to any changes in Rubisco concentration. The effects of drought stress on Rubisco concentration depend on plant species. In this study, reduced *A_sat_* could be due to the decrease in Rubisco activity or the Rubisco activation state [48] which merit our further studies.

### 3.4. Leaf Traits, Shoot and Root Productivities and Water Status

Modification of leaf morphology, such as reductions in leaf number, total leaf area and SLA, is a common drought-avoidance strategy used by plants to reduce the area of transpiration and thus to reduce water loss during drought stress [49]. In this study, prolonged NSIs did not affect leaf thickness as much as leaf initiation (Figure 6A) and expansion (Figure 6B) as all plants had similar SLA (Figure 6C). Limitations of leaf initiation and expansion under drought stress could also explain the decreases in the shoot FW and DW of Tuscan kale grown with prolonged NSIs (Figure 7A,B). This is corroborated by findings from Farooq et al. [50] that drought stress reduced biomass accumulation due to the inhibition of leaf initiation and expansion. Furthermore, drought stress induced by prolonged NSIs impacted the root morphology of Tuscan kale more than its biomass accumulation. This finding was consistent with the works of Tátrai et al. [51] that thyme plants under drought stress invested in a well-developed root system rather than a large root system to increase root absorption capacity. These findings explain why prolonged NSIs only resulted in a small reduction in shoot WC (Figure 7D) without affecting the root WC. Although there were only small decreases in shoot WC in 30 minNSI and 60 minNSI plants compared to 5 minNSI plants (Figure 7D), the lower leaf turgor could have occurred in prolonged NSI plants, which could have resulted in limited water availability for cell expansion [51]. Although there was no statistical difference observed, the reduction in shoot/root ratio FW (Figure 7C) and DW in prolonged NSI plants indicated the use of morphological drought avoidance mechanisms [49]. Thus, Tuscan kale could survive long-term drought stress induced by prolonged NSI by sacrificing vegetative development to maximize water uptake.

Deficit irrigation or mild drought stress can be induced prior to harvest to manage water use more efficiently and enhance crop quality [22]. By doing so, there is less penalty on yield as compared to growing the crops under constant drought stress for the whole life cycle [29]. Hence, one week of drought stress treatment was induced by changing the NSI to a longer duration and the effects on productivity and crop quality of Tuscan kale were studied. It was shown that during the 7-day transfer period, a prolonged NSI from 5 to 90 min (5 minNSI → 90 minNSI) had a negative impact on both shoot and root FW as there was hardly any increase in these two parameters. However, a penalty on the shoot and root biomass accumulation was not seen in 5 minNSI → 60 minNSI plants over the same NSI transfer period (Figure 8A,B). It was found that 5 minNSI → 60 minNSI plants had significantly lower shoot/root ratio FW (Figure 8C) on day 7 after NSI transfer, due to the higher accumulation of root biomass (Figure 8B). Changes in morphology traits such as decreased shoot/root ratio or increased root/shoot ratio in plants during deficit irrigation could have improved water and mineral nutrient uptake during the nutrient spraying period [52].

### 3.5. Nutritional Quality

Phytochemicals such as proline, TSS, total ASC and TPC are the major components attributed to nutritional quality of vegetables which are beneficial for consumers. In response to drought stress, plants can accumulate proline and TSS for osmotic adjustment [25,26]. Proline also has an antioxidant role. It is greatly accumulated in plants under abiotic stress, especially drought and oxidative stresses [32,53]. Although proline is not considered a nutritionally essential amino acid for humans, we have recently witnessed increasing interest in research on the important role of proline in protein synsthesis and its nutrition [54]. Compared to 5 minNSI plants, a higher concentration of proline was observed in 30 minNSI and 60 minNSI plants after transplanting for 4 weeks (Figure 9A), indicating that these plants may have experienced drought stress [25,26]. However, there were no significant differences among NSI treatments for TSS concentration (Figure 9B). Drought stress would trigger an increase in the production of ASC to protect plant cell from oxidative damage [27]. This was supported by the fact that 30 minNSI plants had significantly higher total ASC than those of 5 minNSI plants (Figure 9C). A high concentration of total ASC in 30 minNSI plants could maintain an ETR (Figure 2B) as high as those of 5 minNSI plants since ASC is involved in the Mehler reaction to regulate the redox state of photosynthetic electron carriers [55]. However, an increase in the total ASC concentration was not observed in 60 minNSI plants, which explained the lower ETR in 60 minNSI plants (Figure 2B). This result suggests that whether drought stress leads to increased production of ASC may depend on the degree of drought stress.

In this study, compared to 5 minNSI plants, TPC was significantly higher in 30 minNSI plants but significantly lower in 60 minNSI plants (Figure 9D). In the literature, the effects of drought stress on the accumulation of TPC depend on the duration of drought stress and also on plant species [55]. Under moderate and severe drought stress conditions, the increment of TPC was more preponderant in *Amaranthus* leafy vegetables [36]. However, TPC concentration decreased in grapevine leaves and roots under long-term drought stress [56]. The current study shows that deficit irrigation induced by prolonging NSIs prior to harvest reduced the penalty of drought stress on shoot and root biomass accumulation in 5 minNSI → 60 minNSI plants. Can nutritional quality be improved in Tuscan kale plants under deficit irrigation before harvest? The results were promising in 5 minNSI → 60 minNSI plants as the concentrations of their proline and TSS increased (Figure 10A,B) during the 7-day NSI transfer period without a substantial yield penalty (Figure 8A). However, there were no increases in the total ASC and TPC concentrations measured from the same plants (Figure 10C,D) which could be a result of a limitation of photosynthesis after transferring plants to longer NSIs as the biosynthesis of ASC and TPC is highly dependent on the newly fixed carbon in the leaves [55,57]. For the 5 minNSI → 90 minNSI plants, the changes in proline and TSS, and TPC concentrations were similar to those of 5 minNSI → 60 minNSI plants (Figure 10). However, there were great penalties in their shoot and root biomass accumulation in 5 minNSI → 90 minNSI plants.

## 4. Materials and Methods

### 4.1. Plant Materials and Experimental Design

After germination, Tuscan kale seedlings were inserted into polyurethane cubes and placed along the corridor under an average PPFD of 180 µmol m^−2^ s^−1^ for 10 days before transplanting. Similar-sized seedlings were transplanted onto aeroponic systems and grown in different nutrient spraying intervals of 5, 30 and 60 min [defined as 5 minNSIs, 30 minNSIs (deficit irrigation) and 60 minNSIs (deficit irrigation)] in the greenhouse. The nutrient solution was pumped through discharge pipes installed at the base of the aeroponics trough. The duration of nutrient spraying was 1 min for all plants. All plants were grown at a constant 28 °C root-zone temperature to achieve maximum shoot productivity [40], while their aerial portions were subjected to fluctuating ambient temperatures (23 to 38 °C), light intensity (260 to 950 µmol m^−2^ s^−1^) and RH (40 to 95%). All plants were supplied with full-strength nutrient solution provided by MEOD Pte Ltd. where a conductivity of 2.0 to 2.4 mS cm^−1^ and pH of 5.0 to 5.5 were maintained. Four weeks after transplanting, some 5 minNSI plants were transferred to 60 and 90 minNSI aeroponic systems (defined as 5 minNSI → 60 minNSI and 5 minNSI → 90 minNSI) to induce pre-harvest deficit irrigation.

### 4.2. Measurements of A_sat_, g_s sat_, C_i_ and T_r_

Fully expanded leaves were randomly selected and used for measurements of *A_sat_*, *g*_s_ *_sat_*, *C_i_* and *T_r_* in the greenhouse from 1200 h–1330 h using the LI-COR Portable Photosynthetic System (LI-64000, Bioscience, South San Francisco, CA, USA) which has an LED light source that supplied a PPFD of 1000 μmol m^−2^ s^−1^. Measurements were taken with an average ambient CO_2_ concentration of 415 ± 5 μmol mol^−1^, a relative humidity of 50% ± 3% and an ambient temperature of 38 ± 1 °C.

### 4.3. Measurements of Midday F_v_/F_m_, ETR, △F/F_m_’ and NPQ

The maximum quantum yield of PS II was measured after 25 days of transplanting as F_v_/F_m_ ratio from dark-adapted leaves (15 min in darkness) using the Plant Efficiency Analyser (Hansatech Instruments, King’s Lynn, UK) according to He et al. [58]. Measurements of the F_v_/F_m_ ratio were taken at midday from leaves, which were attached to the plants’ stems in the greenhouse. New fully expanded leaves were also harvested at 0900 h on day 28 after transplanting to determine △F/F_m_’, ETR and NPQ from dark-adapted leaves (15 min in darkness) using the IMAGING PAM MAXI (Walz, Effeltrich, Effeltrich, Germany) in the laboratory. The images of fluorescence emission under different actinic lights were digitized and transmitted via an ethernet interface (GigEVision^®^) to a personal computer for storage and analysis. The △F/F_m_’, ETR, and NPQ were then calculated as described by He et al. [58].

### 4.4. Measurements of Chl and Car Concentrations

Four weeks after transplanting, two pieces of 1 cm diameter leaf discs were harvested and weighed before adding 5 mL of N,N-dimethylformamide (N,N-DMF, Sigma Chemical Co., St. Louis, MO, USA). Pigment samples were placed in the dark for 48 h at 4 °C. The absorption of Chl a, b and Car were measured using a spectrophotometer (UV-2550 Shimadzu, Japan) at 647 nm, 664 nm and 480 nm, respectively, and subsequently calculated using the method described by Welburn [59].

### 4.5. Measurements of NO_3_^−^ and TRN Concentration

Dried leaf tissues of 0.01 g were grounded with 10 mL deionized water before incubating at 37 °C for two hours. Sample turbidity was then removed by vacuum filtering the mixture through a 0.45 µm-pore-diameter membrane. The flow injection analyzer (Model Quikchem 800, Lachat Instruments Inc., Milwaukee, WI, USA) was used to determine the NO_3_^−^ concentration by the catalytic reduction of NO_3_^−^ to nitrite (NO_2_^−^) when the sample passed through a copperized cadmium column. NO_2_^−^ was diazotized with sulphanilamide and coupled with N-(1-naphthyl) ethylenediamine dihydrochloride resulting in a magenta water-soluble dye that was read at 520 nm. The TRN concentration was determined by Kjeldahl digestion of 0.05 g of dried samples and a Kjeldahl tablet in 5 mL of concentrated sulphuric acid for 60 min at 350 °C. After digestion, the TRN concentration was quantified by Kjeltec 8400 analyzer (Foss Tecator AB, Höganäs, Sweden) through titration.

### 4.6. Measurements of TSP and Rubisco Protein Concentrations

To measure the TSP concentration, fresh leaves of 1 g were grounded to a fine powder with liquid nitrogen. A total of 6 mL of extraction buffer [100 mM Bicine-KOH (pH 8.1), 20 mM MgCl_2_, 2% PVP buffer] was added to the powder and mixed thoroughly. The mixture was then centrifuged at 35,000 rpm for 30 min at 4 °C (Beckman ultracentrifuge Optima XL-100K). After centrifugation, 1 mL of the supernatant was mixed with 4 mL of 80% cold acetone and centrifuged for 10 min at 4000 rpm. The precipitate was dissolved in 1 mL of 1 M NaOH before determining the TSP concentration based on an assay described by He et al. [32]. To determine the Rubisco protein content, a protein extract of 500 μL was diluted with 500 μL of solubilizing solution [20% glycerol, 0.02% bromophenolblue, 5% SDS, 0.125 M Tris and 10% β-mercaptoethanol] and boiled for 5 min. After samples were cooled, 10 μL aliquots were loaded into a pre-cast gradient gel (PROTEAN TGX precast gel, BIO-RAD, Hercules, CA, USA). Electrophoresis was performed under a constant voltage of 200 V for 30 min. Separated proteins were then stained for 30 min in coomassie brilliant blue [0.2% coomassie brilliant blue in 10% acetic acid, 50% methanol] and destained with 7% acetic acid and 25% ethanol. Resultant bands were analyzed using a Fluor Chem 8800 gel imaging system under visible light.

### 4.7. Measurements of Leaf Growth, Shoot and Root Productivity and Shoot WC

Four weeks after transplant (day 0 of changing NSIs), five plants from each NSI treatment were harvested and recorded for their numbers of leaves, shoot and root FW. The leaves, stem and roots were then separated and dried at 80 °C for four days before their DW was weighed. The same process was repeated two and seven days after transferring 5 minNSI plants to 60 minNSIs and to 90 minNSIs, to monitor the shoot and root productivity after plants were grown under deficit irrigation at pre-harvest. The total leaf area of the youngest fully expanded leaves was measured using a leaf area meter (WinDIAS3 Image Analysis system). The SLA was estimated as leaf area (cm^2^)/leaf DW (g) while shoot WC was estimated as (FW − DW)/FW.

### 4.8. Measurements of Proline, TSS, Total ASC and TPC Concentrations

Details for the determinations of proline, TSS, total ASC and TPC concentrations were described by He et al. [32].

To determine the proline concentration, frozen leaf tissue of 0.5 g was grounded with 6 mL of 3% sulfosalicylic acid and centrifuged at 9000 rpm for 10 min at 4 °C. A total of 1 mL of supernatant was mixed with equal volumes of acid-ninhydrin and acetic acid before the mixture was heated in a 95 °C water bath for an hour before cooling in an ice bath. The reaction mixture was further extracted with 2 mL of toluene followed by 30 s of vortexing. The absorbance was read at 520 nm using the UV-2550 spectrophotometer (Shimadzu, Japan). TSS was extracted from 10 mg of dried plant tissue with 80% ethanol and the TSS concentration was determined colorimetrically at 490 nm using a spectrophotometer.

To extract ASC, frozen leaf tissues of 0.5 g were grounded to powder with liquid nitrogen together with 1 g of NaCl before the addition of 5 mL ice-cold 2% (*w*/*v*) metaphosphoric acid. The homogenate was centrifuged at 9000 rpm for 30 min at 4 °C. An aliquot of 0.3 mL was mixed with 0.2 mL 45% (*w*/*v*) K_2_HPO_4_ and 0.1 mL 0.1% (*w*/*v*) homocysteine to reduce dehydroascorbic acid to ASC. After 15 min of incubation at 25 °C, 1 mL of 2 M citrate–phosphate buffer (pH 2.3) and 1 mL 0.003% (*w*/*v*) 2,6-dichlorophenolindophenol were added. The ASC was spectrophotometrically assayed at 524 nm. The TPC was extracted from 0.5 g of frozen leaf tissues using 80% methanol and the TPC concentration was measured using a colorimetric method.

### 4.9. Statistical Analysis

The homogeneity of variances was tested using Levene’s test on MINITAB (MINITAB Inc., State College, PA, USA Release 19, 2019). One-way analysis of variances (ANOVA) and Tukey’s multiple comparison tests were then carried out to discriminate between the means of the different groups, where *p* < 0.05 indicated that the means were significantly different. If variances across samples in different NSI treatments were unequal, Welch’s ANOVA and Games-Howell posthoc test were used instead.

## 5. Conclusions

Growing Tuscan kale aeroponically with prolonged NSIs (deficit irrigation) for 4 weeks negatively affected the final yield but enhanced nutritional quality, indicated by increased proline, total ASC and TPC concentrations in 30 minS = NSI plants. Reduced biomass accumulation of Tuscan kale grown with deficit irrigation was mainly caused by the inhibition of leaf initiation and expansion. Although plants grown under deficit irrigation had lower *A_sat_* and *g_s sat_*, all plants had similar *C_i_* and *T_r_*. These results indicate that the reduction of photosynthesis was not attributed to stomatal limitation, but rather to the reduction in photosynthetic light-use efficiency while maintaining constant *T_r_* to moderate leaf temperature in the tropical greenhouse. However, deficit irrigation at pre-harvest by transferring plants grown with 5 minNSIs to 60 minNSIs increased not only the proline but also the TSS concentrations without yield penalty. The amount of energy and water saved through deficit irrigation at pre-harvest can bring substantial benefits to both consumers and growers.

## Data Availability

Raw data used for this study can be found in the NIE Data Repository (https://researchdata.nie.edu.sg/dataverse/he-jie) upon request.
